# Survival and function in elderly patients with GBM: the role of surgical resection with contemporary multimodal therapy

**DOI:** 10.1007/s11060-026-05459-w

**Published:** 2026-03-17

**Authors:** Richard Song, Mark Dapash, Pouya Jamshidi, Vinai Gondi, Rimas V. Lukas, Osaama H. Khan

**Affiliations:** 1 Department of Neurological Surgery, Northwestern University Feinberg School of Medicine, Chicago, IL USA; 2 Department of Pathology, Northwestern University Feinberg School of Medicine, Chicago, IL USA; 3 Department of Radiation Oncology, Northwestern University Feinberg School of Medicine, Chicago, IL USA; 4 Department of Neurology, Northwestern University Feinberg School of Medicine, Chicago, IL USA; 5 Lou and Jean Malnati Brain Tumor Institute at Northwestern Medicine, Chicago, IL USA

**Keywords:** Glioblastoma, Elderly, Survival, Resection, ECOG

## Abstract

**Purpose:**

Is surgical resection associated with better overall survival and preserved functional outcomes in elderly patients with GBM compared to biopsy alone in a single-institution with homogeneous clinical protocols?

**Methods:**

We conducted a single-surgeon, single-center retrospective cohort study of patients aged ≥ 70 years treated between 2016 and 2024 to compare survival following resection versus biopsy for lobar GBM.

**Results:**

Among 82 patients, 65 (79.3%) underwent resection and 17 (20.7%) biopsy. Median survival was significantly longer after resection than biopsy (8.8 vs. 2.7 months). On multivariate Cox analysis adjusting for radiation, temozolomide, MGMT status, and ASA score, resection remained associated with improved survival (HR = 0.34, 95% CI = 0.19–0.63, *p* < 0.001). The survival benefit persisted in both MGMT-methylated and unmethylated tumors but was observed only among patients receiving adjuvant therapy. In octogenarians, resection was independently associated with improved survival (HR = 0.28, 95% CI = 0.10–0.81, *p* = 0.018), with greatest benefit in MGMT-methylated tumors and those treated with temozolomide. Among patients alive with recorded ECOG assessments, 63.6%, 69.2%, and 70.8% had ECOG < 2 at 3, 6, and 12 months, respectively. Nine patients (11%) survived beyond two years; all achieved gross total resection, had MGMT-methylated tumors, and completed full adjuvant therapy.

**Conclusions:**

By applying uniform clinical protocols, these findings support consideration of maximal safe resection in carefully selected elderly GBM patients when combined with multimodal treatment. Given the retrospective design and limited subgroup sizes, however, these findings should be considered exploratory and warrant validation in larger cohorts.

**Supplementary Information:**

The online version contains supplementary material available at 10.1007/s11060-026-05459-w.

## Introduction

Glioblastoma IDH-wildtype (GBM) is the most common primary malignant brain tumor in adults, carrying a median overall survival (OS) of 9 months [1]. Current standard-of-care consists of maximal safe resection followed by postoperative radiation therapy (RTx), chemotherapy, and potentially tumor treating fields [2, 3]. The median age at diagnosis is 65 years, and incidence increases with age, peaking between 70 and 74 years [1].

Reduced tolerance for aggressive therapy in elderly patients with GBM may influence treatment decisions [4–8]. The ANOCEF trial represents the only prospective randomized study comparing resection with biopsy in elderly GBM patients [9]. While OS did not differ between groups (9.37 months for resection vs. 8.96 months for biopsy), extent of resection (EOR) was associated with improved progression-free survival and quality of life. Most retrospective studies outlined in Supplemental Table 1 demonstrate that resection is associated with a significant OS benefit compared to biopsy in elderly GBM patients (median OS 6–15 months for resection, 3–8 months for biopsy). Additionally, higher baseline functional status, receipt of adjuvant RTx and temozolomide (TMZ), and O6-Methylguanine-DNA Methyltransferase (MGMT) methylation have been associated with greater OS. However, all retrospective studies are limited by selection bias; patients with higher baseline functional are more likely to undergo resection, whereas frailer patients are preferentially treated with biopsy and supportive care [10, 11].

Several gaps remain in the literature. First, the majority of studies have a liberal definition of “elderly,” meaning patients older than 65. There are significant increasing risks with age in elderly GBM patients, particularly in octogenarians [12–17], and also tumor and non-tumor intrinsic factors which impact the aggressiveness of the disease [18, 19]. Among the studies that analyzed octogenarians, all find that resection and adjuvant chemoradiation result in better OS in carefully selected patients [11, 13, 17]. However, the heterogeneity in adjuvant therapy regimen [13] and lack of tumor molecular data including isocitrate dehydrogenase (IDH) and MGMT [11, 17] in these studies complicates interpretation.

In addition, functional outcomes following surgery in elderly GBM patients remain poorly characterized. Of the studies that have examined postoperative function, results are conflicting. Klingenschmid and colleagues reported that biopsy and resection patients had similarly good functional outcomes (median KPS 90) three to six months postoperatively [20]. In contrast, Fogg and others observed functional decline in 88% of patients three months after surgery (median biopsy KPS 50, median resection KPS 60) [13]. Whether such discrepancies reflect differences in patient selection, surgical technique, or institutional protocols remain unclear.

To address these gaps, we conducted a single-surgeon, single-center retrospective cohort study of elderly patients who underwent surgery for newly diagnosed GBM between 2016 and 2024. We hypothesized that resection would be associated with greater OS and functional outcomes compared to biopsy in elderly patients, and that this benefit would be modulated by tumor molecular features and adjuvant therapy. We further aimed to determine whether this survival advantage persists in octogenarians.

## Methods

### Study design

This study was conducted in accordance with the Declaration of Helsinki and approved by the Northwestern University Institutional Review Board (NU 16C07 WR: Nervous System Tumor Bank), which permitted analysis and publication of all clinical variables under approved informed consent. We conducted a single-surgeon, single-center retrospective cohort study of patients aged ≥ 70 who underwent gross total resection or biopsy for newly diagnosed GBM between 2016 and 2024 at Northwestern Medicine Central DuPage Hospital. All surgical procedures were performed by the senior author (O.H.K.). Postoperative RTx was administered at the Northwestern Medicine Cancer Center, and chemotherapy was provided by neuro-oncologists at the Northwestern Medicine Warrenville Cancer Center. We defined ‘elderly’ as age ≥ 70 years to focus on an older cohort in whom treatment tolerance and competing comorbidities most strongly influence surgical and adjuvant therapy decisions. Notably, this age cutoff also aligns with the threshold used in the prospective ANOCEF trial [9].

Choice of resection versus biopsy was made by the treating neurosurgeon in conjunction with the multidisciplinary team and patient via shared decision-making. In borderline cases, resection was favored when imaging suggested a high likelihood of achieving maximal safe resection with acceptable neurologic risk; biopsy was favored when expected resection extent was limited, surgical risk was high (e.g., poor functional reserve/comorbidity burden), or based on patient preference.

Patients were excluded if they exhibited radiographic features consistent with gliomatosis cerebri, multifocal disease, butterfly gliomas, and thalamic or deep gray matter involvement as resection is not indicated in such cases, precluding unbiased comparison between biopsy and resection. Individuals with recurrent GBM or a history of prior resection were also excluded.

### Data collection

A retrospective chart review was performed for all patients. Extracted variables included demographics (age, sex, race), tumor molecular markers (IDH mutation and MGMT methylation status), EOR (biopsy vs. resection) from postoperative radiology report, American Society of Anesthesiologists (ASA) score, pre and postoperative ECOG (Eastern Cooperative Oncology Group Performance Status) score, and receipt of postoperative RTx and/or TMZ. OS was defined as the time from surgery to death or last follow-up.

Preoperative ECOG was retrospectively determined from review of hospital admission notes. Scores were assigned using standardized criteria: patients described as fully ambulatory with minor symptoms were classified as ECOG 0–1. Those requiring considerable assistance with ambulation or exhibiting significant fatigue limiting normal activities were classified as ECOG ≥ 2. Because preoperative ECOG status was not prospectively recorded, it was not included in the primary multivariate model to limit bias. Instead, preoperative ASA score, which was prospectively determined by the anesthesiologist and captures baseline comorbidity burden, was used to adjust for baseline health status. Notably, postoperative ECOG was determined by the neuro-oncologist at all follow-up visits.

### Statistical analysis

All analyses were conducted using Python version 3.9.23 with the lifelines library for survival analysis. Descriptive statistics summarized patient demographics and clinical characteristics, stratified by age group (70–74, 75–79, and 80 + years).

Kaplan-Meier survival curves were generated to estimate OS, with log-rank tests used for univariate comparisons between biopsy and resection groups. Stratified univariate analyses were performed by MGMT methylation status, receipt of postoperative RTx therapy, and receipt of at least one cycle of TMZ.

Multivariate Cox proportional hazards regression was performed to evaluate the association between EOR and OS while adjusting for MGMT methylation status, ASA score, and receipt of adjuvant RTx and TMZ. Hazard ratios (HRs) with 95% confidence intervals (CIs) were calculated. Statistical significance was defined as *p* < 0.05. For all variables included in the multivariate Cox models, the proportional hazards assumption was assessed using Schoenfeld residuals, and collinearity was assessed using variance inflation factors (VIF).

## Results

### Patient characteristics

82 patients aged ≥ 70 years underwent surgery for newly diagnosed GBM between 2016 and 2024. The cohort was stratified into three age groups: 70–74 years (*n* = 35, 42.7%), 75–79 years (*n* = 20, 24.3%), and ≥ 80 years (*n* = 27, 32.9%). 65 patients (79.3%) underwent gross total resection; 17 patients (20.7%) underwent biopsy alone. Median OS for patients undergoing resection was 8.8 months, compared to 2.7 months for patients receiving just biopsy. Full patient characteristics are summarized in Table 1.

**Table 1:** Patient characteristics

Characteristic	70–74 yo (*n* = 35)	75–79 yo (*n* = 20)	80 + yo (*n* = 27)	Total (*n* = 82)
**Demographics**				
Median age (range)	72 (70–74)	76.5 (75–79)	83 (80–91)	75 (70–91)
Gender, n (%)	25 Male (71.4%)	12 Male (60.0%)	14 Male (51.9%)	51 Male (62.2%)
Preop ASA Score				
2	2 (5.7%)	2 (10.0%)	1 (3.7%)	5 (6.1%)
3	31 (88.6%)	16 (80.0%)	18 (66.7%)	65 (79.3%)
4	2 (5.7%)	1 (5.0%)	8 (29.6%)	11 (13.4%)
Preop ECOG Score				
0	14 (40.0%)	6 (30.0%)	6 (22.2%)	26 (31.7%)
1	18 (51.4%)	14 (70.0%)	14 (51.9%)	46 (56.1%)
2	3 (8.6%)	0 (0.0%)	7 (25.9%)	10 (12.2%)
**Tumor Characteristics**				
Extent of Resection, n (%)				
Biopsy	3 (8.6%)	6 (30.0%)	8 (29.6%)	17 (20.7%)
Gross Total Resection	32 (91.4%)	14 (70.0%)	19 (70.4%)	65 (79.3%)
MGMT status, n (%)				
Methylated	16 (45.7%)	9 (45.0%)	11 (40.7%)	36 (43.9%)
Unmethylated	19 (54.3%)	11 (55.0%)	16 (59.3%)	46 (56.1%)
IDH status, n (%)				
Wild Type	32 (91.4%)	20 (100.0%)	25 (92.6%)	77 (93.9%)
Unknown	3 (8.6%)	0 (0.0%)	2 (7.4%)	5 (6.1%)
**Adjuvant Therapy**				
Received RTx, n (%)	30 (85.7%)	13 (65.0%)	12 (44.4%)	55 (67.1%)
Days from surgery to RT, median (range)	30.5 (8–69)	22 (6–42)	32.5 (18–48)	32 (6–48)
RTx regimen, n (%)				
60 Gy / 30 fractions	16 (53.3%)	5 (38.5%)	0 (0.0%)	21 (38.2%)
40 Gy / 15 fractions	7 (23.3%)	6 (46.2%)	10 (83.3%)	23 (41.8%)
Other	7 (23.3%)	2 (10.0%)	2 (7.4%)	11 (13.4%)
Received TMZ ≥ 1 cycle, n (%)	21 (60.0%)	8 (40.0%)	13 (48.1%)	42 (51.2%)
Received Avastin, n (%)	12 (34.3%)	3 (15.0%)	8 (29.6%)	23 (28.0%)
Received Tumor Treating Fields, n (%)	7 (20.0%)	3 (15.0%)	2 (7.4%)	12 (14.6%)
**Survival**				
Median months (range)	10.8 (0.3–51.2)	4.2 (0.3–16.5)	5.4 (0.2–36.4)	6.5 (0.2–51.2)
Median months by resection (range)				
Biopsy	1.7 (0.3–2.7)	3.3 (0.3–16.5)	3.0 (0.2–8.5)	2.7 (0.2–16.5)
Resection	11.3 (0.53–51.2)	5.4 (0.7–16.1)	6.5 (0.7–36.4)	8.8 (0.5–51.2)

### Overall survival in all elderly patients

**Fig. 1:**
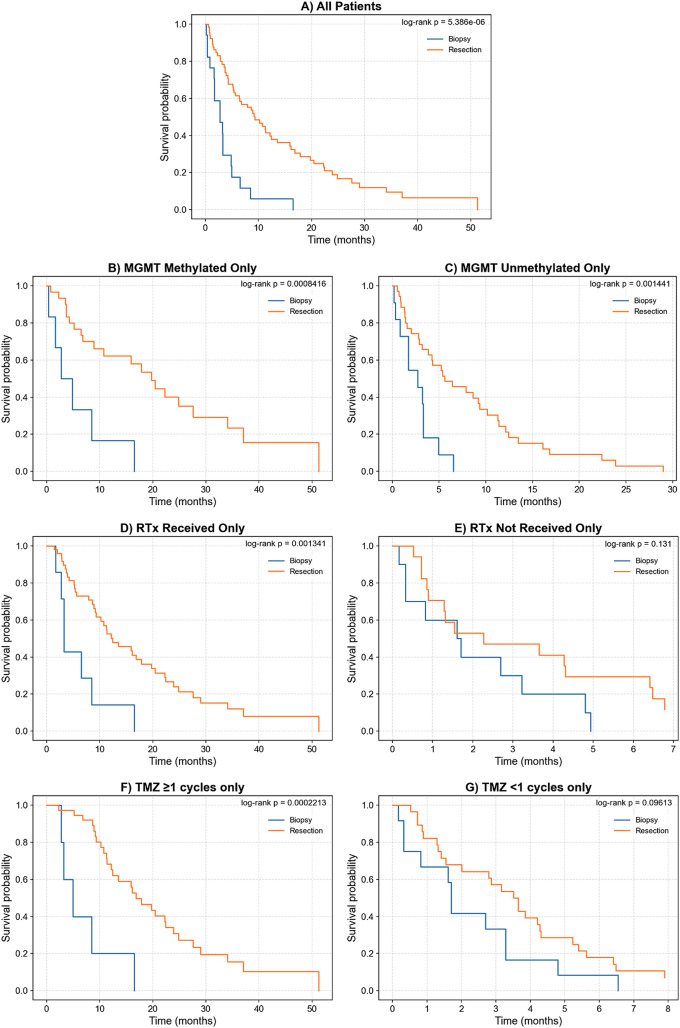
Kaplan-Meier curves comparing GBM extent of resection (biopsy or resection) on overall survivorship, with univariate log-rank p-values quantifying statistical significance. Survivorship analysis was done on **A**) all patients in our cohort, **B**) patients who were MGMT methylated, and **C**) patients who were MGMT unmethylated **D**) patients who received RTx, **E**) patients who did not receive RTx, **F**) patients who received at least one cycle of adjuvant TMZ chemotherapy, **G**) patients who did not receive any adjuvant TMZ

Median OS for the entire cohort was 6.5 months. Patients who underwent resection had longer median OS compared with those who underwent biopsy alone (8.8 vs. 2.7 months, log-rank *p* = 5.39E-06; Fig. 1A). This survival benefit persisted when stratified by MGMT methylation status, with significant advantages in both MGMT-unmethylated (log-rank *p* = 8.42E-04) and MGMT-methylated subgroups (log-rank *p* = 1.44E-03; Fig. 1B and C). Results of univariate analyses examining associations between clinical variables and OS are detailed in Supplemental Table 2.

The survival benefit of resection was significant among patients who received RTx (log-rank *p* = 1.34E-03; Fig. 1D) but not in those who did not receive RTx (log-rank *p* = 0.13; Fig. 1E). Similarly, resection was associated with improved OS in patients who received TMZ (log-rank *p* = 2.21E-04; Fig. 1F), but not in those who received no TMZ (log-rank *p* = 0.096; Fig. 1G).

On multivariate Cox proportional hazards regression adjusting for MGMT methylation, ASA score, receipt of RTx, and TMZ treatment, resection remained independently associated with significantly improved OS (HR 0.34, 95% CI 0.19–0.63, *p* = 6.34E-04; Table 2). Other significant predictors of improved OS included MGMT methylation (HR 0.29, 95% CI 0.16–0.52, *p* = 4.17E-05), RTx (HR 0.24, 95% CI 0.12–0.48, *p* = 6.18E-05), and TMZ receipt (HR 0.14, 95% CI 0.07–0.27, *p* = 8.74E-09). The proportional hazards assumption was not violated (Supplemental Table 3), and VIFs were < 4 for all covariates, indicating no evidence of problematic collinearity (Supplemental Table 4).

### Survival in octogenarians (Age 80+)

**Fig. 2:**
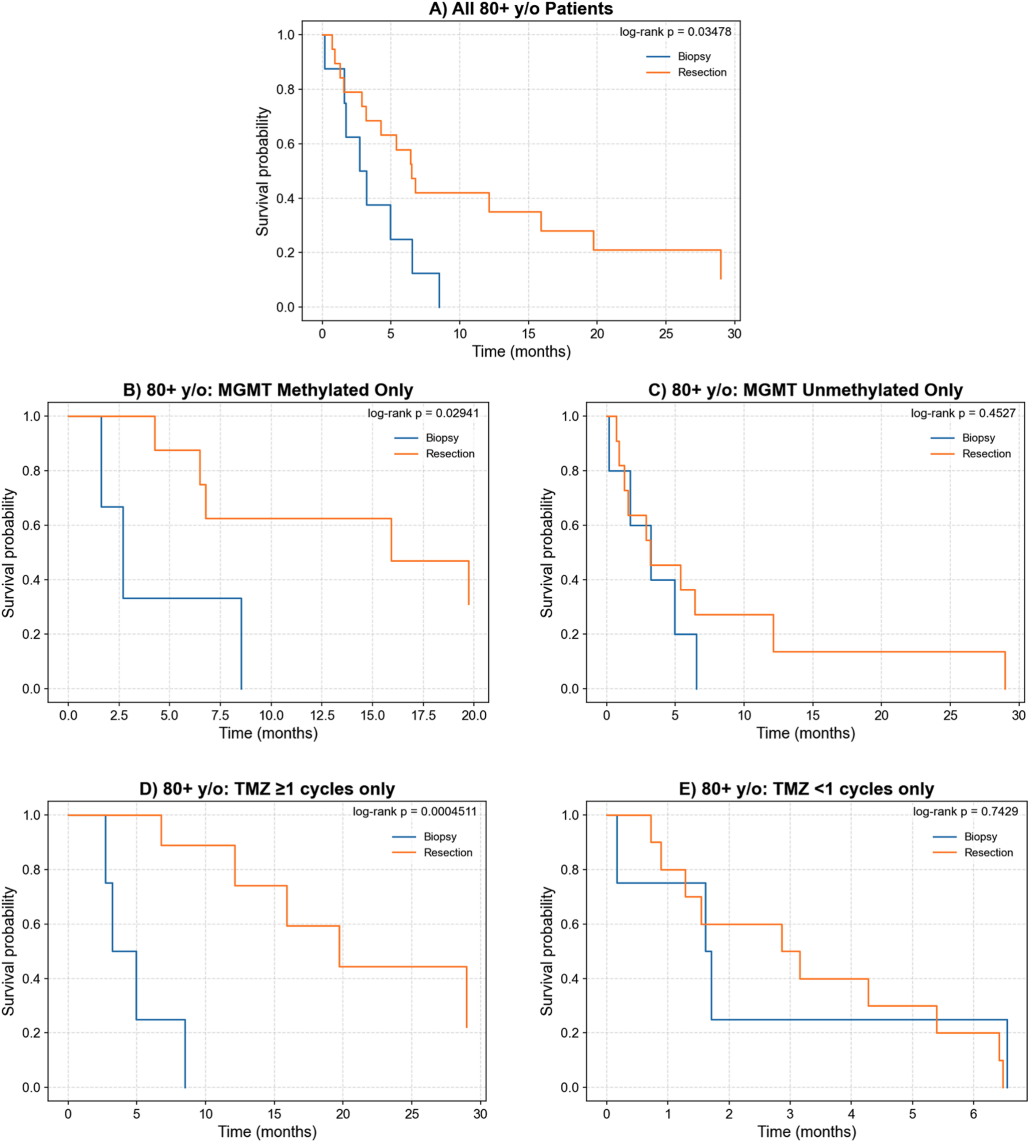
Univariate survivorship analysis in octogenarians (≥ 80 years old). Kaplan–Meier curves are shown for all patients (**A**), stratified by MGMT methylated (**B**) and unmethylated (**C**) tumors, and stratified by those who received at least one adjuvant TMZ cycle (**D**) versus those who did not (**E**)

Among the 27 patients aged ≥ 80 years, median OS was 5.4 months (range: 0.2–36.4 months). Patients who underwent resection experienced longer median OS than those treated with biopsy (6.5 vs. 3.0 months, log-rank *p* = 0.035; Fig. 2A).

On multivariate Cox regression analysis adjusting for MGMT methylation, RTx, TMZ, and ASA, resection was independently associated with significantly improved OS in octogenarians (HR 0.28, 95% CI 0.10–0.81, *p* = 0.018; Table 2). Other independent predictors of improved OS included RTx (HR 0.13, 95% CI 0.04–0.47, *p* = 1.81E-03) and TMZ receipt (HR 0.09, 95% CI 0.04–0.20, *p* = 6.67E-10). MGMT methylation had a marginally significant improvement in OS (HR = 0.43, 0.18–1.01, *p* = 0.053). The proportional hazards assumption was not violated (Supplemental Table 3), and VIFs were < 3 for all covariates, indicating no evidence of problematic collinearity (Supplemental Table 4).

Among octogenarians with MGMT-methylated tumors, resection was associated with significantly improved survival compared to biopsy (log-rank *p* = 0.029; Fig. 2B), but no benefit was observed in those with MGMT-unmethylated tumors (log-rank *p* = 0.45; Fig. 2C). A significant survival advantage was observed among patients who received TMZ (log-rank *p* = 4.51E-04, Fig. 2D), but no difference was observed in those who did not receive TMZ (log-rank *p* = 0.74, Fig. 2E). EOR was not significantly associated with survival among octogenarians who received RTx (log-rank *p* = 0.21) or those who did not receive RTx (log-rank *p* = 0.20). Of the 12 octogenarians who received RTx, only two underwent biopsy, limiting statistical power in this subgroup.

**Table 2:** Multivariate cox regression analysis of overall survival

Variable	All Patients (70+)			Octogenarians (80+)		
	HR	95% CI	*p*-value	HR	95% CI	*p*-value
Resection (vs. biopsy)	0.34	0.19–0.63	6.34E-04	0.28	0.10–0.81	0.018
MGMT methylation	0.29	0.16–0.52	4.17E-05	0.43	0.18–1.01	0.053
RTx Received	0.24	0.12–0.48	6.18E-05	0.13	0.04–0.47	1.81E-03
TMZ (≥ 1 cycle)	0.14	0.07–0.27	8.74E-09	0.09	0.04–0.20	6.67E-10
ASA score ≥ 4	0.91	0.36–2.23	0.84	0.7	0.24–2.01	0.50

To assess effect modification by MGMT status, an interaction term (resection × MGMT) was included in the multivariate Cox model for octogenarians. The magnitude of benefit of resection appeared more pronounced in MGMT-methylated tumors (HR 0.09) than in unmethylated tumors (HR 0.46), and this interaction reached marginal statistical significance (p-interaction = 0.051).

### Survival in patients with good preoperative functional status

To address selection bias, we performed an analysis stratified by preoperative ECOG. Among patients with good baseline performance (ECOG 0–1), 64 (88.9%) underwent resection and 8 (11.1%) underwent biopsy. In contrast, among patients with ECOG 2, 2 (20%) underwent resection, whereas 8 (80%) underwent biopsy. We then compared OS between biopsy and resection patients only in the preoperative ECOG 0–1 group. In univariate Kaplan-Meier analysis, resection remained associated with significantly improved OS compared with biopsy (log-rank *p* = 0.005) (Supplemental Fig. 1). In multivariate Cox proportional hazards analysis within this subgroup, receipt of RTx (HR = 0.28, *p* = 1.80E-03), MGMT methylation (HR = 0.26, *p* = 5.97E-05), and treatment with at least one cycle of TMZ (HR = 0.11, *P* = 8.19E-7) were associated with improved OS, whereas the association between EOR and OS was not statistically significant (HR = 0.60, *p* = 0.12).

### Post- operative functional status

We evaluated postoperative functional outcomes using ECOG performance scores recorded at neuro-oncology follow-up visits. Analyses were restricted to patients who were alive at each timepoint and had ECOG documented and therefore reflect conditional functional status among survivors. Detailed results are shown in Table 3. Among survivors with available functional assessments, the majority maintained good performance status (ECOG 0–1) at each timepoint. At 3 months postoperatively, 65.3% of all resection patients and 75.0% of octogenarian resection patients had ECOG 0–1. This favorable distribution persisted at 6 months (69.4% and 80.0%, respectively) and at 1 year (70.0% and 71.4%, respectively). Across all evaluated time points, the distribution of ECOG scores did not differ significantly between the resection and biopsy groups (Fisher’s exact test, all *p* > 0.05).

**Table 3:** Serial postoperative functional status outcomes

All Patients:									
	3 m			6 m			1 yr		
	Resect	Biopsy	Total	Resect	Biopsy	Total	Resect	Biopsy	Total
**ECOG Category (n%):**									
ECOG 0	4 (8.2%)	0 (0.0%)	4 (7.3%)	4 (11.1%)	0 (0.0%)	4 (10.3%)	3 (13.0%)	0 (0.0%)	3 (12.5%)
ECOG 1	28 (57.1%)	3 (50.0%)	31 (56.4%)	21 (58.3%)	2 (66.7%)	23 (59.0%)	13 (56.5%)	1 (100.0%)	14 (58.3%)
ECOG 2	10 (20.4%)	2 (33.3%)	12 (21.8%)	9 (25.0%)	0 (0.0%)	9 (23.1%)	6 (26.1%)	0 (0.0%)	6 (25.0%)
ECOG 3	6 (12.2%)	0 (0.0%)	6 (10.9%)	2 (5.6%)	1 (33.3%)	3 (7.7%)	1 (4.4%)	0 (0.0%)	1 (4.2%)
ECOG 4	1 (2.0%)	1 (16.7%)	2 (3.6%)	0 (0.0%)	0 (0.0%)	0	0 (0.0%)	0 (0.0%)	0 (0.0%)
**Survival Status (n%)**									
Alive, ECOG Recorded	49 (94.2%)	6 (75.0%)	55 (91.7%)	36 (90.0%)	3 (100.0%)	39 (90.7%)	23 (95.8%)	1 (100.0%)	24 (96.0%)
Alive, ECOG Not Recorded	3 (5.8%)	2 (25.0%	5 (8.3%)	4 (10.0%)	0 (0.0%)	4 (9.3%)	1 (4.2%)	0 (0.0%)	1 (4.0%)
Total Alive	52	8	60	40	0	43	24	1	25
**Median ECOG [IQR]**	1 [1–2]	1.5 [1–2]	1 [1–2]	1 [1–2]	1 [1–3]	1 [1–2]	1 [1–2]	1 [1–1]	1 [1–2]
**ECOG < 2**	32 (65.3%)	3 (50.0%)	35 (63.6%)	25 (69.4%)	2 (66.7%)	27 (69.2%)	16 (70.0%)	1 (100.0%)	17 (70.8%)
**ECOG ≥ 2**	17 (34.7%)	3 (50.0%)	20 (36.4%)	11 (30.6%)	1 (33.3%)	12 (30.8%)	7 (30.0%)	0 (0.0%)	7 (29.2%)

Octogenarians (80+):									
	3 m			6 m			1 yr		
	Resect	Biopsy	Total	Resect	Biopsy	Total	Resect	Biopsy	Total
**ECOG Category**									
ECOG 0	3 (25.0%)	0 (0.0%)	3 (20.0%)	3 (30.0%)	0 (0.0%)	3 (25.0%)	2 (28.6%)	0 (0.0%)	2 (28.6%)
ECOG 1	6 (50.0%)	2 (66.7%)	8 (53.3%)	5 (50.0%)	1 (50.0%)	6 (50.0%)	3 (42.9%)	0 (0.0%)	3 (42.9%)
ECOG 2	3 (25.0%)	1 (33.3%)	4 (26.7%)	2 (20.0%)	0 (0.0%)	2 (16.7%)	2 (28.6%)	0 (0.0%)	2 (27.6%)
ECOG 3	0 (0.0%)	0 (0.0%)	0 (0.0%)	0 (0.0%)	1 (50.0%)	1 (8.3%)	0 (0.0%)	0 (0.0%)	0 (0.0%)
ECOG 4	0 (0.0%)	0 (0.0%)	0 (0.0%)	0 (0.0%)	0 (0.0%)	0 (0.0%)	0 (0.0%)	0 (0.0%)	0 (0.0%)
**Survival Status**									
Alive, ECOG Recorded	12 (85.7%)	3 (75.0%)	15 (83.3%)	10 (90.9%)	2 (100.0%)	12 (92.3%)	7 (100.0%)	0 (0.0%)	7 (100.0%)
Alive, ECOG Not Recorded	2 (14.3%)	1 (25.0%)	3 (16.7%)	1 (9.1%)	0 (0.0%)	1 (7.7%)	0 (0.0%)	0 (0.0%)	0 (0.0%)
Total Alive	14	4	18	11	2	13	7	0	7
**Median ECOG [IQR]**	1 [1–2]	1 [1–2]	1 [1–2]	1 [0.5–1.5]	2 [1–3]	1 [1–2]	1 [0–2]	N/A	1 [0–2]
**ECOG < 2**	9 (75.0%)	2 (66.7%)	11 (73.3%)	8 (80.0%)	1 (50.0%)	9 (75.0%)	5 (71.4%)	0 (0.0%)	5 (71.4%)
**ECOG ≥ 2**	3 (25.0%)	1 (33.3%)	4 (26.7%)	2 (20.0%)	1 (50.0%)	3 (25.0%)	2 (28.6%)	0 (0.0%)	2 (28.6%)

### Long-term survival profiles

Nine patients (11% of the resection cohort) survived beyond two years, including two octogenarians (Supplemental Table 5). All long-term survivors underwent resection and had ECOG 0–1 preoperatively. 8 of 9 (89%) harbored MGMT promoter methylated tumors. The MGMT unmethylated patient exhibited chromosome 10 loss, likely resulting in absence of functional MGMT expression despite an unmethylated promoter. All long-term survivors completed concurrent chemoradiation followed by six cycles of adjuvant TMZ. In addition, three patients (33%) received tumor-treating fields (TTF) and three (33%) were treated with bevacizumab. Most patients maintained ECOG 1–2 one year postoperatively. There was no dominant tumor location observed among long-term survivors.

## Discussion

Our findings add to the evidence supporting that maximal safe surgical resection and adjuvant management of GBM in elderly patients may be associated with greater OS [4, 5, 21–25]. Our series represents one of the largest single-center cohorts of elderly GBM patients undergoing resection, with uniform surgical technique and postoperative management. Prior studies have suggested a benefit of resection but were often limited by heterogeneity in surgical approaches and adjuvant protocols [4, 9, 11, 13, 26–29] or incomplete molecular profiling, with many lacking MGMT or IDH data [5, 11, 14, 17, 22, 27, 29, 30]. By incorporating molecular markers and adjuvant treatment into multivariate models, our study suggests that the survival benefit of resection persists even after accounting for these variables.

To address selection bias, we reviewed patients with good preoperative functional status (ECOG 0–1). Within this subgroup, resection remained associated with improved OS on univariate analysis; however, after multivariate adjustment, the independent effect of resection was no longer statistically significant, whereas MGMT methylation status and receipt of adjuvant therapy remained strong predictors of outcome. Although this analysis should be interpreted cautiously given retrospective ECOG assignment and the small number of biopsy patients with good baseline functional status, it suggests that the observed survival advantage is not solely attributable to selection of healthier patients for resection but rather reflects the combined benefit of resection and adjuvant therapy in appropriately selected patients.

Stratified analyses of the overall cohort demonstrated a survival benefit of resection irrespective of MGMT methylation status. In contrast, within octogenarians, a significant survival advantage was observed only in patients with MGMT-methylated tumors, suggesting that tumor biology may exert a greater influence on the therapeutic value of resection in octogenarians [19]. Although limited by sample size, this is, to our knowledge, the first study to examine the effect of EOR stratified by MGMT status in octogenarians.

We also found that the survival advantage associated with resection in the overall cohort was evident only when followed by adjuvant therapy. These findings align with prior reports suggesting that the benefit of resection is maximized when integrated with comprehensive multimodal therapy. For example, Niare et al. reported a median OS of 17.5 months among octogenarians treated with resection followed by the Stupp protocol, compared with 9.5 months among all resected patients [11]. Our findings also contextualize the ANOCEF trial, which reported no OS difference between resection and biopsy in elderly patients [9]. In that study, patients enrolled before 2017 received radiotherapy alone, whereas those enrolled later received combined chemoradiotherapy, introducing temporal confounding that may have attenuated any survival advantage of resection – consistent with our observation that surgical benefit is most apparent when both RTx and TMZ are delivered.

A major contribution of this study is the assessment of postoperative functional trajectories. Among resection patients who survived to follow-up, the majority maintained functional independence, with ECOG 0–1 in 65.3% at 3 months, 69.4% at 6 months, and 70.0% at 1 year. Similar proportions were observed in octogenarians. These findings contrast with those of Fogg et al. [13], who reported functional decline in 88% of octogenarians at 3 months, but are more consistent with Klingenschmid et al. [20], who reported preserved function at 3–6 months with median KPS of 90 in both groups. Our analysis of long-term survivors further supports functionally meaningful survival: eight of nine patients surviving beyond two years, including two octogenarians, maintained ECOG 1–2 at one year, underwent resection, completed full adjuvant therapy, and had excellent baseline functional status. Given the relatively small sample size of patients who were long-term survivors and had serial postoperative ECOG measurements, these results should also be interpreted as exploratory. However, our data suggest that, in carefully selected elderly patients, resection can extend quality survival.

There are several limitations to our study. Although the single-surgeon approach reduces technical variability, it may not reflect outcomes across different surgical practices. The modest sample size, particularly among octogenarians, restricts statistical power for subgroup and interaction analyses. In particular, the small biopsy subgroups raise concerns about model stability; therefore, subgroup and interaction results should be interpreted as exploratory and not definitive. Although we adjusted for ASA score, MGMT methylation, and adjuvant therapy, we lacked validated measures of baseline frailty and cognitive status; therefore, unmeasured differences in patient fitness and tumor biology may contribute to residual confounding. In addition, functional outcomes were assessed at routine follow-up visits and are therefore conditional on survival and visit completion; differential mortality and missing ECOG assessments may bias comparisons of postoperative function between treatment groups. For all of these reasons, the results can only be interpreted as associative and not causative. Finally, because our cohort was restricted to patients ≥ 70 years, findings may not directly generalize to ‘younger elderly’ patients (65–69 years) included in some prior studies.

Future studies with larger sample sizes should validate these findings across more heterogeneous populations. Integration of molecular and genomic profiling may further refine patient selection [24]. Prospective studies incorporating patient-reported outcomes and longitudinal functional trajectories will be essential to balance OS against quality-of-life considerations. Future directions could also verify whether supramarginal resection may result in significant survival benefits over gross total resection in elderly patients with GBM. Overall, our work supports that maximally safe resection ought to be considered as a component of a multimodal treatment plan for GBM in elderly patients.

## Supplementary Information

Below is the link to the electronic supplementary material.

Supplementary Material 1

## Data Availability

The datasets generated during and/or analyzed during the current study are available from the corresponding author on reasonable request.

## Abbreviations

GTR: Gross Total Resection
STR: Subtotal Resection
RTx: Radiation Therapy
TMZ: Temozolomide
ECOG: Eastern Cooperative Oncology Group
IDH: Isocitrate Dehydrogenase
MGMT: O6-methylguanine-DNA methyltransferase
OS: Overall Survival
GBM: Glioblastoma
TTF: Tumor Treating Fields
ASA: American Society of Anesthesiologists
