# Supplementary material for: Survival and function in elderly patients with GBM: the role of surgical resection with contemporary multimodal therapy
Source: J Neurooncol. 2026 Mar 17;177(2):62. doi: 10.1007/s11060-026-05459-w (PMC12995949; doi:10.1007/s11060-026-05459-w)
Supplement: Supplementary file 1 — Supplementary Material 1 [file 11060_2026_5459_MOESM1_ESM.docx]

## Supplemental Data

Supplemental Table 1. Previous Literature Assessing Surgical Outcomes in Elderly GBM Patients.

| **Study** | **Year** | **Study Design** | **Age Criteria** | **N** | **Treatment Groups** | **Key Findings** | **Key Limitations** | **Survival (Median unless otherwise specified)** |
| --- | --- | --- | --- | --- | --- | --- | --- | --- |
| Chaichana et al. [4] | 2011 | Retrospective | > 65 years (mean 73 ± 5 years) | 129 | Resection: 129 (100%) | Lower KPS, motor deficit, language deficit, cognitive deficit, and tumor size larger than 4 cm were associated with lower survivorship | Stupp protocol not standard practice at the time of this study. No reference matched group of biopsy patients with similar preoperative characteristics to compare the effects of EOR. No quality of life measures reported postoperatively. | Overall: 7.9 months |
| Abdullah et al. [21] | 2015 | Retrospective | ≥80 years (mean 83, range 80-93) | 52 | GTR: 12 (21%)  STR: 40 (69%) | Higher preoperative KPS, postop adjuvant therapy, and lack of p53/EGFR expression improved survival. EOR not associated with improved survival | Relatively limited GTR and sample size. Patients enrolled from 2003 - 2009 did not get p53/EGFR screening. No quality of life measures reported postoperatively. | Overall: 4.2 months  No adjuvant therapy: 3.2 months  Radiation only: 6.6 months  Radiation + Chemo: 11.5 months |
| Hoffermann et al. [22] | 2015 | Retrospective | >65 years (mean 71, range 65-84) | 125 | GTR: 35 (28.0%)  STR: 28 (22.4%)  PR: 34 (27.2%)  Biopsy: 27 (21.6%) | Larger EOR, high baseline KPS, and receipt of the Stupp protocol were all factors associated with longer survival | Limited octogenarian population (n = 7) and no mention of how MGMT or IDH affect survivorship. No quality of life measures reported postoperatively. | GTR: 15.0 months  STR: 11.0 months  PR: 6.4 months  Biopsy: 5.6 months |
| Heiland et al. [23] | 2018 | Retrospective | ≥65 years (median 73.4, IQR 9.28 years) | 342 | Resection: 216 (63.2%)  Biopsy: 125 (36.5%) | GTR improved OS compared to biopsy. Higher preop KPS associated with improved survival. Combined radio-chemotherapy beneficial, particularly in MGMT-negative GBM. Postop KPS for resection was higher than biopsy (80 vs 70) | Only 50% of patients had reported MGMT status. The time at which postop KPS was assessed was not discussed. | GTR: 10.8 months  Overall cohort: 7.5 months |
| Pessina et al. [24] | 2018 | Retrospective | ≥65 years (median 71, range 65-83) | 178 | Complete resection: 8 (4.5%)  GTR: 63 (35.4%)  STR: 46 (25.8%)  PR: 16 (9.0%)  Biopsy: 45 (25.3%) | All patients treated with surgery + RTx + TMZ. Tumor location, EOR, and postop neuro status affected survival. | Large treatment heterogeneity between 2004 - 2015 when this study took place. No functional outcome or quality of life data | Overall: 12.2 months  GTR: 15.1 months  STR: 11.9 months  PR: 8 months  B: 8.1 months |
| Karsy et al. [5] | 2018 | Retrospective | ≥75 years (mean 80.5 ± 3.8 years) | 82 | GTR: 19 (23.2%)  STR: 33 (40.2%)  Biopsy: 18 (22.0%)  No surgery: 8 (9.8%) | Increased EOR was associated with longer overall survival only in patients that did not have complications. Post-op KPS was lower in patients who had complications and patients who lived less than 12 months | Modest sample size of patients who underwent GTR (n = 19), and did not consider how adjuvant therapy status or tumor molecular markers affected survivorship. The time when postop KPS was assessed was not specified | Overall:  3.0 +/- 1.0 months (median)  6.3 ± 1.2 months (mean)  Mean survivals by EOR:  No surgery: 0.8 ± 0.3 months  Biopsy: 3.7 ± 1.1 months  Subtotal resection: 5.0 ± 1.4 months  Gross-total resection: 12.1 ± 3.0 months |
| Glynn et al. [14] | 2019 | Retrospective | ≥70 years (Range 70-87 years) | 104 | Debulked: 55 (52.9%)  Biopsy: 49 (47.1%) | Biopsy only, Higher ECOG, and increasing age were associated with statistically significant shorter survival | Functional outcomes after surgery were not assessed. The association between aggressive/palliative treatment and tumor molecular markers (MGMT) on survivorship were not assessed. | Debulked: 8.0 months  Biopsy: 4 months |
| Schwartz et al. [25] | 2020 | Retrospective | ≥65 years (mean 73.1 ± 5.1) | 160 | Resection: 100% | Increased EOR did not correlate with survival. Age and KPS remained strongest predictors. | No biopsy comparison group. High complication rates (10% surgery complications, 30% treatment related complications). Functional outcomes not assessed postoperatively. | Overall: 10 months |
| Lopez-Rivera et al. [26] | 2021 | Database | ≥65 years (median 73) | 17820 | GTR: 14%  STR: 28%  Biopsy: 42% | GTR and STR associated with improved survival | Treatment heterogeneity across multiple institutions in the database. Lack functional outcome data (KPS, QOL), detailed surgical variables (EOR, complications), and molecular markers (MGMT often missing). | Not specified |
| Klingenschmid et al. [20] | 2022 | Retrospective | ≥65 years (mean 74, range 65-88) | 121 | GTR: 74 (61.2%)  STR: 20 (16.5%)  Biopsy: 27 (22.3%) | GTR offers the greatest OS compared to STR and biopsy. KPS for GTR, STR, and biopsy groups remain similar postoperatively. Good KPS in 3-6 month follow-up (median 90) overall. | No information about survivorship or functional status based on adjuvant therapy status. No analysis of how molecular markers of tumors affected survivorship. Limited analysis of 80+ year old patients (n=18) | GTR: 13.8 months  Biopsy: 4.0 months |
| Niare et al. [17] | 2022 | Retrospective | ≥80 years (mean 82) | 117 | Resection: 57 (48.7%)  Biopsy: 60 (51.3%) | Resection group had better baseline WHO PS, better ASA scores, more right-sided tumors. Patients receiving resection + Stupp protocol had best outcomes. | Limited MGMT or IDH data pre 2021. Resection group consisted of 19 multifocal tumors (33.3%) which is typically not considered to be resectable. Functional outcomes and quality of life not assessed. | Resection: 9.5 months (95% CI: 8-17)  Biopsy: 4 months (95% CI: 3.5-6)  Resection + Stupp: 17.5 months (95% CI: 12-24) |
| Li et al. [27] | 2022 | Database and Retrospective | >=75 years (median 77.2 +/- 2.93 at author hospital) | Author Hospital: 50  Database: 6079 | Author Hospital:  GTR: 39 (78%)  STR: 7 (14%)  PR: 4 (8%)  Database:  GTR: 1598 (26.2%)  STR: 1570 (25.8%)  Biopsy: 1176 (19.3%)  No surgery: 1735 (28.5%) | GTR and STR have favorable prognosis. GTR was best protective factor of OS | Small sample size in author hospital, especially those who got GTR. Treatment heterogeneity across multiple institutions in database. Lack functional outcome data (KPS, QOL) | Author Hospital:  Partial resection: 2.7 months  STR: 6.6 months  GTR: 9.1 months  Database:  GTR: 6 months  STR: 4 months  Biopsy: 3 months  No surgery: 2 months |
| Bruno et al. [28] | 2022 | Retrospective | >65 years | 135 | GTR: 10 (7.4%)  STR: 102 (75.6%)  Biopsy: 17.0%) | Large extent of resection (EOR), MGMT  methylation, baseline KPS >= 70, and receipt of adjuvant chemoradiation  therapy were predictors of longer overall survival | Limited by the small number of patients (n = 10) who underwent GTR as well as patients older than 80 years old (n = 9) | GTR: 32.8 months  STR: 9.9 months  Biopsy: 6.0 months |
| Fogg et al. [13] | 2023 | Retrospective | ≥80 years (mean 83.6, range 80-96) | 123 | Biopsy: 65 (52.8%)  Resection: 58 (47.2%) | GTR, TMZ, Pre-op KPS > 70, and MGMT associated with better survival. Most patients (88%) declined in functional status 3 months postop | Long term functional status outcomes not discussed. Large heterogeneity with radiation therapy protocols (only 21% received standard radiation, 28% had unknown dose, 51% received "abbreviated courses"). GTR significantly predicted OS in multivariate testing even though EOR didn't in univariate testing: the potential confounding effects are not discussed | Biopsy alone: 1.6 months  Resection, TMZ, Radiation: 7.5 months |
| Baumgarten et al. [10] | 2023 | Retrospective | ≥75 years (median 79, range 75-110) | 143 | Resection: 39 (27.3%)  Biopsy: 104 (72.7%) | KPS ≥70, surgery vs biopsy, adjuvant chemo, and adjuvant RT all significantly associated with OS in univariate analysis. In multivariate analysis, only radiation and chemo associated with OS, but not EOR | No assessment of functional outcomes after surgery. | Overall: 5.9 months  Surgery: 12 months  Biopsy: 5 months  KPS ≥70: 6 months vs KPS ≤60: 3 months |
| Laigle-Donadey et al. [9] | 2023 | Prospective | ≥70 years | 101 | Resection: 50 (50%)  Biopsy: 51 (50%) | Resection had greater PFS than biopsy, but no difference in OS. Biopsy associated with more severe QOL impairment | Patients enrolled during 2008-2017 were not given temozolomide chemotherapy, as that was not standard practice. Limited average cases per hospital per year leads to clinical heterogeneity. Most patients enrolled had low baseline KPS (<=70) | Resection: 9.37 months  Biopsy: 8.96 months |
| Horowitz et al. [29] | 2024 | Database | >65 years | 27090 | Not specified | GTR, RT, and chemo associated with improved OS in octogenarians | Treatment heterogeneity across multiple institutions in database. Lack functional outcome data (KPS, QOL), detailed surgical variables (EOR, complications), and molecular markers (MGMT often missing). | Overall: 6 months |
| Stadler et al. [17] | 2024 | Retrospective | ≥80 years (mean 82) | 107 | Biopsy: 45 (45%)  STR: 30 (30%)  GTR: 25 (25%) | GTR is associated with better OS outcomes than biopsy, but no difference between GTR and STR. Higher baseline KPS and treatment beyond surgery also associated with better outcomes. | Lack functional outcome data after surgery. Many molecular marker data, including IDH and MGMT, are missing. | Overall OS: 4.2  OS if received postop treatment: 7.2 months |

Supplemental Table 2. Univariate Analysis of Features Associated with OS

|  | **All Patients** | | | **Octogenarians** | | |
| --- | --- | --- | --- | --- | --- | --- |
| **Variable** | **HR** | **95% CI** | **p value** | **HR** | **95% CI** | **p value** |
| Age | 1.03 | 0.99-1.08 | 0.11 | 1.01 | 0.88-1.14 | 0.93 |
| Gender | 0.92 | 0.47-1.49 | 0.74 | 0.57 | 0.23-1.41 | 0.22 |
| MGMT Methylation | 0.35 | 0.21-0.58 | 5.78E-05 | 0.39 | 0.16-0.95 | 0.039 |
| ASA Score ≥ 4 | 1.78 | 0.91-3.49 | 0.094 | 1.18 | 0.48-2.91 | 0.71 |
| RTX Received | 0.21 | 0.12-0.38 | 1.45E-07 | 0.25 | 0.086-0.71 | 9.42E-03 |
| TMZ (≥1 cycle) | 0.11 | 0.06-0.21 | 5.22E-12 | 0.1 | 0.026-0.35 | 3.67E-03 |
| Tumor Treating Fields (Optune) | 0.99 | 0.53-1.86 | 0.98 | 2.64 | 0.58-11.98 | 0.21 |
| Avastin | 0.66 | 0.39-1.11 | 0.12 | 0.82 | 0.33-2.00 | 0.66 |
| Tumor Size (Max Dimension) | 1.04 | 0.89-1.21 | 0.59 | 1.00 | 0.78-1.29 | 0.97 |

Supplemental Table 3. Proportional Hazards Assumption Checks (Schoenfeld residuals)

|  | **All Patients** | | **Octogenarians** | |
| --- | --- | --- | --- | --- |
| **Variable** | **Rank Trans p-value** | **KM Trans p-value** | **Rank Trans. p-value** | **KM Trans. p-value** |
| Resection (vs. biopsy) | 0.54 | 0.54 | 0.32 | 0.34 |
| MGMT Methylation | 0.74 | 0.74 | 0.071 | 0.076 |
| RTX Received | 0.61 | 0.64 | 0.52 | 0.52 |
| TMZ (≥ 1 cycle) | 0.45 | 0.47 | 0.80 | 0.80 |
| ASA Score ≥ 4 | 0.74 | 0.74 | 0.29 | 0.30 |

Rank Trans = Rank Transformation; KM Trans = Kaplan Meier Transformation

Supplemental Table 4. Variance Inflation Factors Values

| **Variable** | **All Patients** | **Octogenarians** |
| --- | --- | --- |
| Resection | 3.45 | 2.27 |
| MGMT Methylation | 1.89 | 2.01 |
| RTx Received | 3.47 | 2.22 |
| TMZ ≥1 Cycle | 2.84 | 2.51 |
| ASA Score ≥ 4 | 1.08 | 1.21 |

Supplemental Table 5. Long-Living Patient Demographics


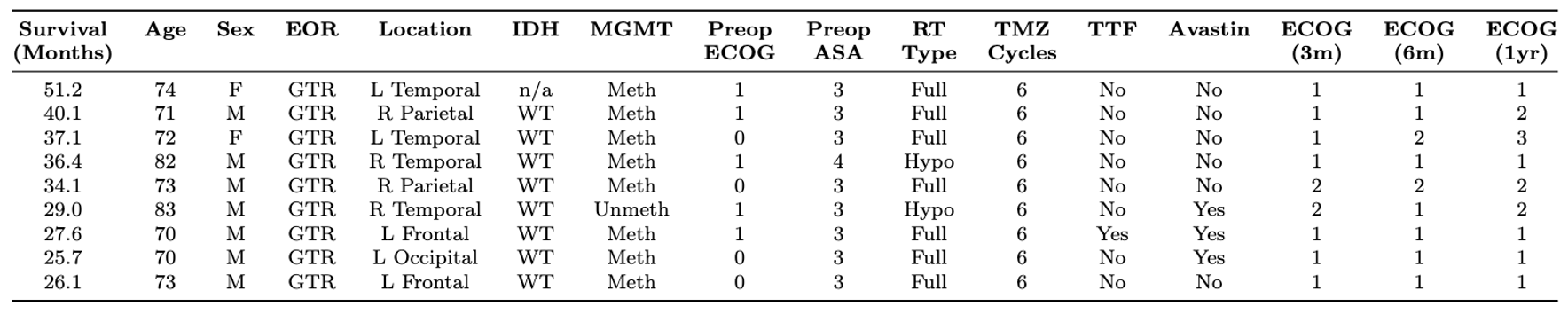


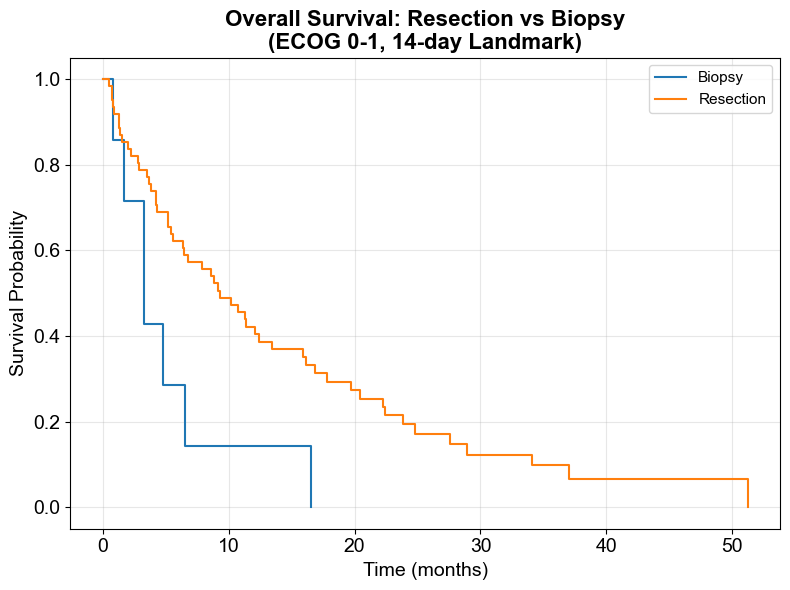


Supplemental Figure 1. Kaplan-Meier survivorship between patients with preoperative ECOG 0-1 who received biopsy or resection who survived at 14 days post-surgery.
